# Irrigation constraints shape the global potential for multiple cropping expansion on existing cropland

**DOI:** 10.1088/1748-9326/ae44ae

**Published:** 2026-03-06

**Authors:** Felicitas D Beier, Jens Heinke, Benjamin Leon Bodirsky, Jan Philipp Dietrich, Kristine Karstens, Sebastian Ostberg, David Meng-Chuen Chen, David Hötten, Pascal Sauer, Gabriel Abrahão, Hermann Lotze-Campen, Alexander Popp, Christoph Müller

**Affiliations:** 1 Potsdam Institute for Climate Impact Research (PIK), Member of the Leibniz Association, Potsdam, Germany; 2 Humboldt-Universität zu Berlin (HU), Albrecht Daniel Thaer-Institut für Agrar- und Gartenbauwissenschaften, Berlin, Germany; 3 Humboldt-Universität zu Berlin, Integrative Research Institute on Transformations of Human-Environment Systems (IRI THESys), Berlin, Germany; 4 University of Kassel, Faculty of Organic Agricultural Sciences, Witzenhausen, Germany

**Keywords:** multiple cropping, irrigation, land use, cropland, global, water availability

## Abstract

Multiple cropping increases land productivity by allowing multiple harvests per year, offering production gains without cropland expansion. Irrigation is especially critical in the seasonally dry tropics, enabling multiple cropping where otherwise only a single rainfed cycle would be feasible. Estimates of the current state of multiple cropping and the multiple cropping expansion potential without changes in irrigation patterns exist, but the multiple cropping expansion potential through irrigation expansion has not yet been assessed at the global scale. Here, we estimate multiple cropping expansion potentials on existing cropland considering the interaction with irrigation and local water availability constraints to determine how much cropland area can be managed in multiple cropping systems and the associated increases in annual yields and crop production. We find that, under current climatic conditions, there is considerable global biophysical potential to expand multiple cropping on existing cropland, particularly when also expanding irrigation. Total global crop production could increase by 28% (from 4 200 mio. t DM to 5 400 mio. t DM). This gain stems from nearly quadrupling the area under rainfed multiple cropping, more than doubling multiple cropping area within already irrigated lands, and expanding irrigation into areas where it facilitates another growing season. Our study reveals a considerable multiple cropping expansion potential on existing cropland that—when tapped—could contribute to averting further cropland expansion to meet future demand for agricultural outputs. Local irrigation water availability constrains the irrigation-enabled multiple cropping potential, implying that the interaction of multiple cropping and irrigation is crucial to consider in comprehensive land and water assessments that account for biophysical and socio-economic constraints, sustainability criteria, and land competition under future global change.

## 1. Introduction

Agricultural systems face increasing pressure as demand for food, feed, bioenergy, and bio-material products grows due to demographic, economic, and climate-related drivers (Shukla *et al* 2019, Stehfest *et al* 2019, Bodirsky *et al* 2020). Multiple cropping—i.e. planting and harvesting a field more than once a year—is widely practiced and enables higher production per physical cropland unit (Mueller *et al* 2012, Ray and Fole 2013, Wu *et al* 2014, Waha *et al* 2020).

Multiple cropping is common in tropical and subtropical regions where two or more growing cycles are climatically feasible and is especially important in cereal production (Siebert *et al* 2010, Waha *et al* 2020, Xu *et al* 2021, Brar *et al* 2022). Globally, 12% of cropland (135 Mha) is multiple cropped, most of it irrigated (Waha *et al* 2020). In South and Southeast Asia, irrigation enables two or three harvests on the same plot and seasonal irrigation water availability is essential for sustaining high cropping intensities (Kitamur 1987, Radulovic 2000, Biemans *et al* 2016, FAO 2022).

Accounting for the interaction of irrigation and multiple cropping is crucial for global irrigation and land system assessments to avoid misrepresenting cropping patterns and irrigation water use in these major producing regions, and to prevent underestimating incentives for irrigation and water stress (Waha *et al* 2020, 2025, Beier *et al* 2023).

Global gridded data sets exist for average cropping intensity (Siebert *et al* 2010) and existing multiple cropping systems (Waha *et al* 2020), based on national and sub-national data. They provide insights into the current multiple cropping extent, showing that multiple cropping often coincides with irrigation. The potential to increase production by increasing the harvesting frequency has also been assessed in previous studies (Mauser *et al* 2015, Wu *et al* 2018, Waha *et al* 2020). For example, Wu *et al* (2018) identify the spatially explicit potential harvest frequency on current cropland for four crops and typical cropping sequences under temperature and precipitation constraints. Mauser *et al* (2015) use a dynamic crop growth model to estimate the potential biomass increase through multiple cropping under current climatic conditions and irrigation patterns. The potential to expand multiple cropping by expanding irrigation, however, has not yet been assessed.

We go beyond previous research (Mauser *et al* 2015, Wu *et al* 2018) by assessing multiple cropping expansion potential on existing cropland under potential irrigation expansion and local water availability constraints. We address the research question: What is the multiple cropping production potential under irrigation expansion and local irrigation water availability constraints on existing cropland? To answer this question, we determine where rainfed and irrigated multiple cropping is feasible, estimate spatially explicit annual yields, irrigation water consumption and withdrawals under single and multiple cropping, identify where irrigation-enabled multiple cropping is possible, and calculate the resulting production increase.

To do so, we developed a method to approximate the multiple cropping suitability, yields and irrigation water requirements under multiple cropping conditions with the outputs of the gridded dynamic global vegetation, crop, and hydrology model LPJmL (von Bloh *et al* 2018). With LPJmL, we simulate (i) the main-growing-season yield for 12 crop types, and (ii) the monthly productivity of managed perennial grass for the whole year. Off-season crop water requirements and yields are derived using the off-season-to-main-season ratio of grass productivity for each crop and its specific growing season. The method is generic enough to be adapted to other crop-model-based workflows, and to be integrated into land system modeling frameworks that currently mostly rely on crop model runs that do not represent multiple growing seasons per crop (Biemans *et al* 2016, Mathison *et al* 2021, Waha *et al* 2025) and instead consider a uniform and constant regional or country-level cropping intensity factor without accounting for the interaction with irrigation (e.g. MAgPIE (Dietrich *et al* 2019, 2025)). As such, our method can contribute to improving the geographical heterogeneity of production patterns and water use in global land and water assessments, especially in tropical regions that rely heavily on irrigation to facilitate another growing season and produce a large share of global agricultural output (Biemans *et al* 2016, Waha *et al* 2020).

## 2. Methods

### 2.1. Scenarios

In this analysis, we assess the potential production increase on existing physical cropland through multiple cropping under consideration of local irrigation water availability constraints during the main and additional growing seasons. For reasons of data availability and consistency, we choose 2010 as reference year. We distinguish three scenarios—the reference state (REF) and two multiple cropping expansion scenarios: one representing the biophysical potential under consideration of water availability constraints (POT) and one theoretical benchmark scenario with no water availability limitation (NWL) – to assess the role of irrigation and local water availability in enabling production increases through multiple cropping expansion (see table 1).

**Table 1.** Description of cropping scenarios.

Scenario name	Scenario description
REF: Reference state	Reference state in 2010, taking the prevailing crop area management into account (i.e. irrigated and rainfed conditions and the share of crop area under single and multiple cropping system as derived from LandInG (Ostberg *et al* 2023)).
POT: Potential (with water limitation)	In this scenario, the biophysical multiple cropping potential on the same physical cropland extent (see REF scenario) is realized taking local water availability constraints into account (Beier *et al* 2023).
NWL: Potential with unlimited water supply (no water limitation)	Hypothetical benchmark scenario in which multiple cropping is expanded to its maximum extent on the same physical cropland extent (see REF scenario) without considering local water availability constraints. Multiple cropping is expanded to all areas that are suitable for multiple cropping (see multiple cropping suitability in figure 2 in SI). It serves as a useful benchmark and comparison point to previous research (e.g. Mauser *et al* (2015)).

To determine the global multiple cropping potential on existing cropland given limited irrigation water availability, we use data on

• the historical spatial distribution of physical cropland extent and harvested area of crops under rainfed and irrigation conditions derived using the Land Input Generator (LandInG) (Ostberg *et al* 2023) (see section 2.2);
• potential multiple cropping suitability under irrigated versus rainfed conditions determined by the method presented here (see section 2.4);
• spatially-explicit single- and multiple-cropping crop yields and the respective crop water requirements derived using the method presented here (see section 2.4) with data from the dynamic global vegetation, crop, and hydrology model LPJmL (von Bloh *et al* 2018) (see section 2.3); and
• local irrigation water availability constraints considering upstream-downstream relationships of the 0.5 ^∘^ grid cells based on the method introduced in Beier *et al* (2023) (see section 2.5).

### 2.2. Global gridded crop area dataset

We obtain crop-specific gridded harvested areas, total physical cropland extent and fallow land at 0.5 ^∘^ spatial resolution using the Land Input Generator (LandInG) (Ostberg *et al* 2023). LandInG harmonizes harvested area information at the country level from Monfreda *et al* (2008), AQUASTAT (FAO 2020), MIRCA 2000 (Portmann *et al* 2010), and FAOSTAT (FAO 2021) and disaggregates it spatially to be consistent with the physical crop area extent of LUH v.2v2 (Hurtt *et al* 2020), using additional data from Monfreda *et al* (2008), Ramankutty *et al* (2008), HYDE v.3.2.1 (Klein Goldewijk *et al* 2017) and assumptions about the climatic suitability for multiple cropping (see section 2.4 and supplementary information (SI) section 1.1).

The resulting data includes information on the spatial extent of rainfed and irrigated crop area management systems in 2010 – both in terms of physical and harvested areas, whereas crop-specific area information is aggregated to 17 crop types (see SI section 1.3). We use the cropping intensity (i.e. the ratio of the harvested area to the physical cropland extent in a grid cell) to determine the area share under multiple cropping for each crop in the grid cell in the reference state (REF) (see also SI section 1.1).

### 2.3. Global gridded biophysical data

Biophysical data (e.g. crop yields, irrigation water requirements) and hydrological data (e.g. runoff) are derived from outputs of the dynamic global vegetation, crop, and hydrology model LPJmL (von Bloh *et al* 2018, Lutz *et al* 2019, Wirth *et al* 2024). LPJmL provides crop-specific (irrigated and rainfed) crop yields and crop evapotranspiration for the main growing season of 12 crop functional types and gross primary productivity (GPP) and evapotranspiration of grass for each month of the year at a 0.5 ^∘^ spatial resolution.

### 2.4. Approximation of multiple cropping suitability, crop yields and water requirements

The location-specific climatic multiple cropping suitability under irrigated and rainfed conditions is determined based on monthly GPP of grass under irrigated and rainfed conditions provided by LPJmL, with unlimited irrigation water supply. Aiming for a parsimonious method that captures climatic conditions that influence crop growth throughout the year, we use monthly grass GPP to determine grid cells that are suitable for multiple cropping. We assume a month to be a ‘growing period month’ if monthly grass GPP exceeds 100 g C m^−2^ (∼2.2 t DM ha^−1^) and classify grid cells as ‘suitable for multiple cropping under irrigated/ rainfed conditions’ if at least nine months fulfill this condition. The parameters are chosen to align multiple cropping patterns to the multiple cropping suitability of GAEZ v.4 for the historical period 2000–2010 (FAO 2022) (see SI section 1.4 for more details).

To quantify the potential additional yield achieved through multiple cropping, we develop a generic metric that approximates the ‘off-season’ yield, i.e. the yield achieved outside the main growing season modeled by LPJmL. LPJmL does not model sequential cropping within one year on the same plot of land explicitly. Crops are only grown in the prescribed growing season (here referred to as ‘main season’) that can differ for rainfed and irrigated growing conditions (Minoli *et al* 2019). Grass growth is, however, modeled throughout the entire year in LPJmL, reflecting the seasonality of growing conditions throughout the year.

Our method to derive multiple cropping yields computes for each modeled LPJmL crop type and its respective growing season an off-season-to-main-season ratio of grass GPP. This ratio is used to scale the crop yield of the explicitly simulated main growing season of the respective crop to estimate the off-season crop yield (see SI section 1.5). It does not explicitly distinguish whether the ‘off-season’ incorporates one or two additional growing cycles, but instead approximates the total productivity that could be gained in months outside the main season. To account for effects that dampen multiple cropping yields, including time between harvest of the first and sowing of the second crop (Hansel *et al* 2019), we reduce off-season yields by 25% compared to the full potential in the off-season (see also SI equation (4)).

In order to assess whether irrigation demand for both seasons can be satisfied, the irrigation water requirements for the main- and off-season are calculated taking LPJmL data on transpiration, evaporation and interception in the main irrigated growing season for all simulated crops and irrigation system assumptions into account. Consumptive irrigation water requirements in the main irrigated growing season are derived from the difference in evapotranspiration under irrigated conditions to evapotranspiration under rainfed conditions in the same (irrigated) growing season and is obtained from the additional transpiration, evaporation, and interception taking the prevalence of different irrigation systems (drip, sprinkler, surface) based on data by Jägermeyr *et al* (2015) into account (see SI section 1.8 for more details).

To obtain off-season consumptive irrigation water requirements, we establish linear regressions between crop and grass irrigation water consumption during the main growing season for each crop-irrigation system combination (see SI figures 4–6 for the relationship between grass and crop consumptive irrigation water requirements). These regression coefficients are then applied to estimate off-season crop water consumption. Total annual irrigation water requirements combine main-season consumptive irrigation water requirements with off-season estimates (adjusted by the same fallow factor used for the estimation of yields) and total irrigation water consumption and withdrawals are derived following assumptions in Rost *et al* (2008), Jägermeyr *et al* (2015), Schaphoff *et al* (2018) regarding irrigation systems and their respective field and conveyance losses (see SI section 1.8 for more details).

### 2.5. Irrigation water availability

Local irrigation water availability is determined using the ‘mrwater’ flow accumulation algorithm that operates at 0.5^∘^ resolution and takes upstream-downstream relationships into account while optimizing the allocation of potentially irrigated areas based on the potential productivity gains through irrigation within each grid cell (see SI section 1.9 and Beier *et al* (2023) for more details). In this algorithm, water resources within a radius of 100 km can be used to fulfill human water demands of the respective grid cell (see SI section 1.9 for more details). Within the upstream-downstream accounting, non-agricultural water uses—provided by Wada *et al* (2016)—are prioritized over irrigation. Already irrigated areas under consideration of multiple cropping patterns as of the REF scenario are fulfilled before allocating additional freshwater resources for irrigation expansion. The consumed fraction by a priority user is not available for withdrawal further downstream, but return flows can be withdrawn further downstream. For cases where water resources are not sufficient to fulfill exogenous water demands (non-agricultural water demand and irrigation water demand of the REF scenario), non-renewable groundwater usage is assumed (see SI section 1.9). Potential expansions of the irrigated areas rely on available renewable water resources within the radius of 100 km and no additional non-renewable groundwater resources can be tapped for expansions. For irrigation expansion, grid cells are ranked by the total yield gain achieved through irrigation including the potential additional yield through multiple cropping, with higher-ranking grid cells within the same river basin served prior to lower-ranking cells. For each grid cell and its prescribed land use (i.e. crop composition as of 2010), total irrigation water demand for the given crop area under multiple cropping are determined. If available freshwater resources are sufficient, irrigation expansion occurs in this grid cell and downstream flows and upstream constraints are updated. If available freshwater resources are insufficient to meet the full irrigation demand, the irrigation expansion area is reduced proportionally across all crops within the grid cell.

### 2.6. Crop production

Using crop area patterns derived using LandInG (section 2.2), yields and multiple cropping expansion potential as calculated using the methodology described above (section 2.4), we calculate annual crop production as the product of annual crop yields and crop area (see SI section 1.7) for the reference state (REF), the potential scenario with multiple cropping expansion under consideration of water availability constraints (POT) and a hypothetical scenario for comparison in which there is no local water availability limitation (NWL), i.e. with unlimited water supply (see 2.1).

To account for country-specific crop management factors that affect the crop yield achieved, we calibrate potential yields derived from LPJmL to country-level yields derived from FAOSTAT production information (FAO 2021) while maintaining within-country spatial patterns from LPJmL (see SI section 1.6).

For a detailed description of the data and methodology refer to section 1 in the SI.

## 3. Results

### 3.1. Potential expansion of multiple cropping is limited by local irrigation water availability

**Figure 1.**
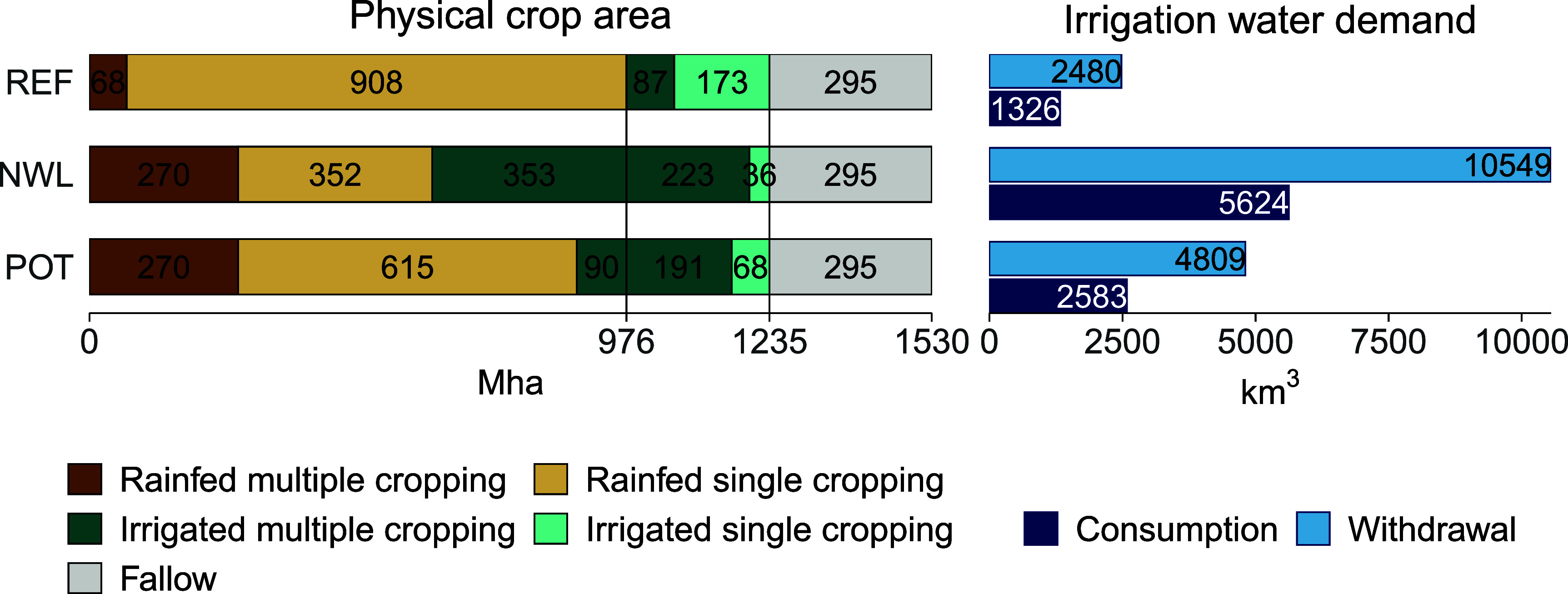
Physical crop area (in Mha) by management system for the scenarios REF (distribution of cropping systems on total physical cropland area in 2010), NWL (multiple cropping expansion potential without consideration of local water availability constraints), POT (multiple cropping expansion potential considering local water availability constraints) and associated total water demand (in km^3^ yr^−1^ ) distinguishing water withdrawals and water consumption. The vertical lines demarcate the extent of rainfed and irrigated cropland in 2010 in the reference state (REF).

Figure 1 shows the physical cropland extent and its management (rainfed, irrigated, single cropped, multiple cropped, fallow) for the reference state (REF), the multiple cropping expansion scenario with no water limitation where multiple cropping can be expanded to all suitable areas (NWL), and the scenario of potential multiple cropping expansion under consideration of local water availability constraints (POT), and associated irrigation water demand.

**Figure 2.**
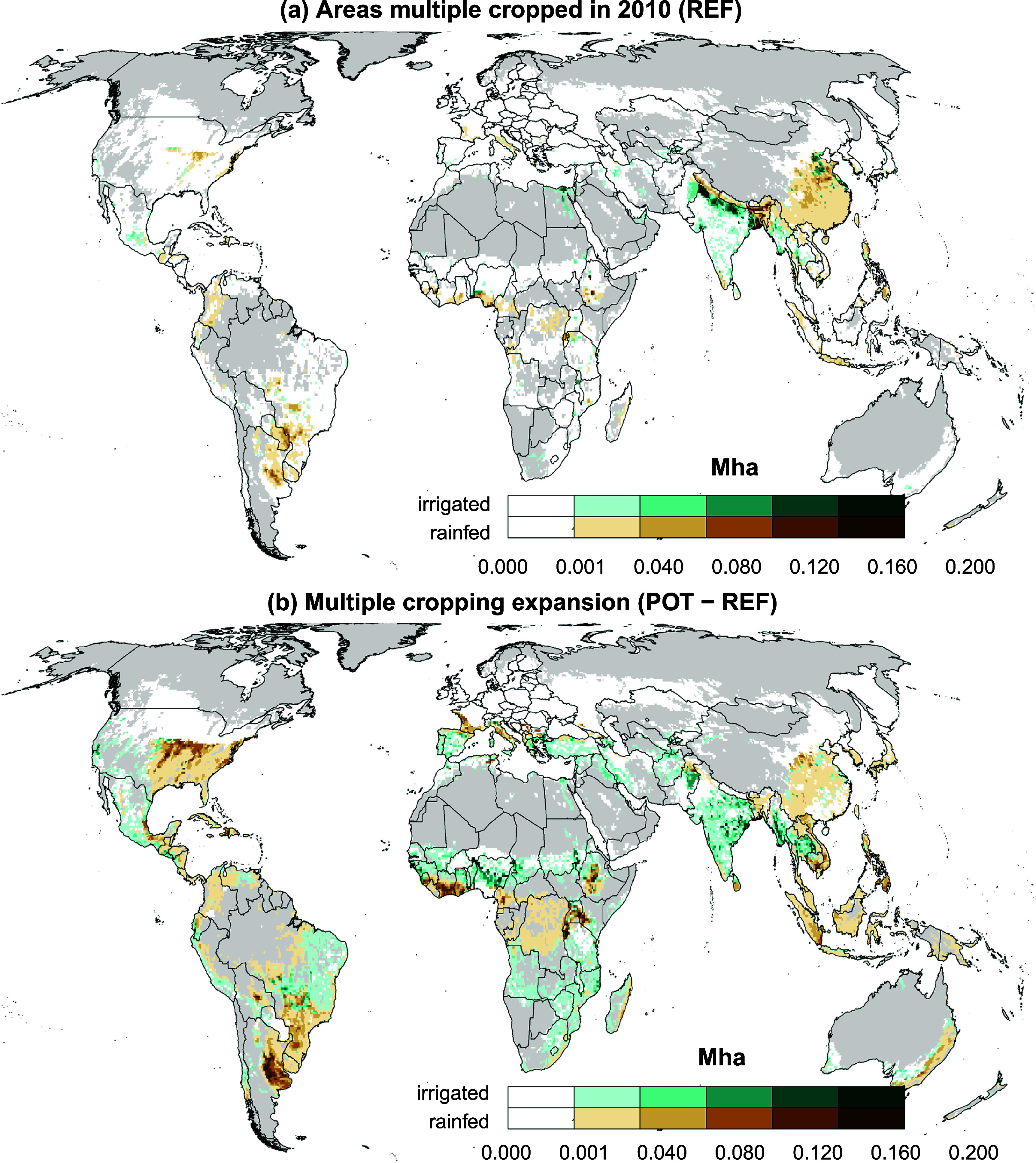
Areas (in Mha) that are multiple cropped (REF, upper panel (a)) or where multiple cropping expansion is possible POT-REF, lower panel (b)).

Total physical cropland extent in 2010 amounts to 1 500 Mha with 295 Mha being left fallow. The remaining 1 235 Mha are under active cropping. Of these, 21% (260 Mha) are irrigated and 79% (976 Mha) are rainfed. Multiple cropping occurs on 7% (68 Mha) of rainfed and 33% (87 Mha) of irrigated cropland. The spatial extent of multiple cropped areas is shown in figure 2(a).

Considering only climatic multiple cropping suitability, rainfed multiple cropping could be expanded to 28% of rainfed cropland (270 Mha) and irrigated multiple cropping could be expanded to almost 580 Mha if no local water availability constraints are considered (NWL). About 220 Mha of which would be located on already irrigated areas (∼85% of irrigated cropland) and roughly 350 Mha are areas where irrigation is required to overcome water limitations that prevent multiple cropping under rainfed conditions (i.e. ‘irrigation-enabled multiple cropping’ on previously rainfed ). However, total global irrigation water demand to irrigate those areas under the respective management system would more than quadruple (withdrawals increase from 2 500 km^3^ yr^−1^ to 10 500 km^3^ yr^−1^ and consumption from 1 300 km^3^ yr^−1^ to 5 600 km^3^ yr^−1^ from REF to NWL). The locations where this multiple cropping expansion under irrigated conditions could take place do not always have enough freshwater resources available to fulfill such high irrigation water demand (see figure 8(c) in SI for spatial extent of these areas with insufficient water for multiple cropping expansion).

Under consideration of local water availability for irrigation (allowing a maximum water transport distance of 100 km), the multiple cropping expansion potential under irrigated conditions reduces to ∼280 Mha – 190 Mha of which are located in areas that are already irrigated and 90 Mha of which would entail irrigation-enabled multiple cropping on previously rainfed areas. The irrigation-enabled multiple cropping area expansion is substantially lower than in the NWL scenario, highlighting the importance of considering water availability constraints in assessments of multiple cropping expansion potentials. Still, on previously irrigated areas, multiple cropping could roughly double from ∼90 Mha to ∼190 Mha expanding multiple cropping to 73% of irrigated areas.

### 3.2. Spatial extent of multiple cropping expansion potential

Figure 2 shows the distribution of multiple cropped areas for the REF and the multiple cropping expansion potential under consideration of local irrigation water availability constraints (*POT-REF*). In REF, rainfed multiple cropping dominates in China, Argentina, Paraguay and Southern Brazil, while irrigated multiple cropping is prevalent in Northern India. An expansion of irrigated multiple cropping is possible in parts of India, South-East Asia, Sub-Saharan Africa, and Brazil. Rainfed multiple cropping can be expanded in the United States, South-East Asia, Central Africa and Southern Brazil.

We also show that while the Mediterranean area, Mexico and additional areas in India and Central Africa would have growing conditions that would allow for further expansion of multiple cropping (NWL, see SI figure 8(b), available freshwater resources are not sufficient to expand multiple cropping in those areas to reach this potential (NWL—POT), SI figure 8(c). This shows the importance of considering hydrological constraints in the assessment of production potentials.

Section 3.2 in the SI provides further details on the multiple cropping expansion extent in terms of cropland shares (see SI figure 9). It highlights China, Paraguay, Argentina, Southern Brazil and Central Africa as regions with multiple cropping expansion potential under rainfed conditions; Mexico, Southern Europe and Turkey as well as South- and South-East Asia and China where an expansion of irrigated multiple cropping on previously irrigated areas is possible; and Sub-Saharan Africa and Eastern Brazil as well as Myanmar and Thailand as regions where the expansion of irrigation would facilitate multiple cropping.

### 3.3. Potential production increases through multiple cropping expansion on existing cropland

**Figure 3.**
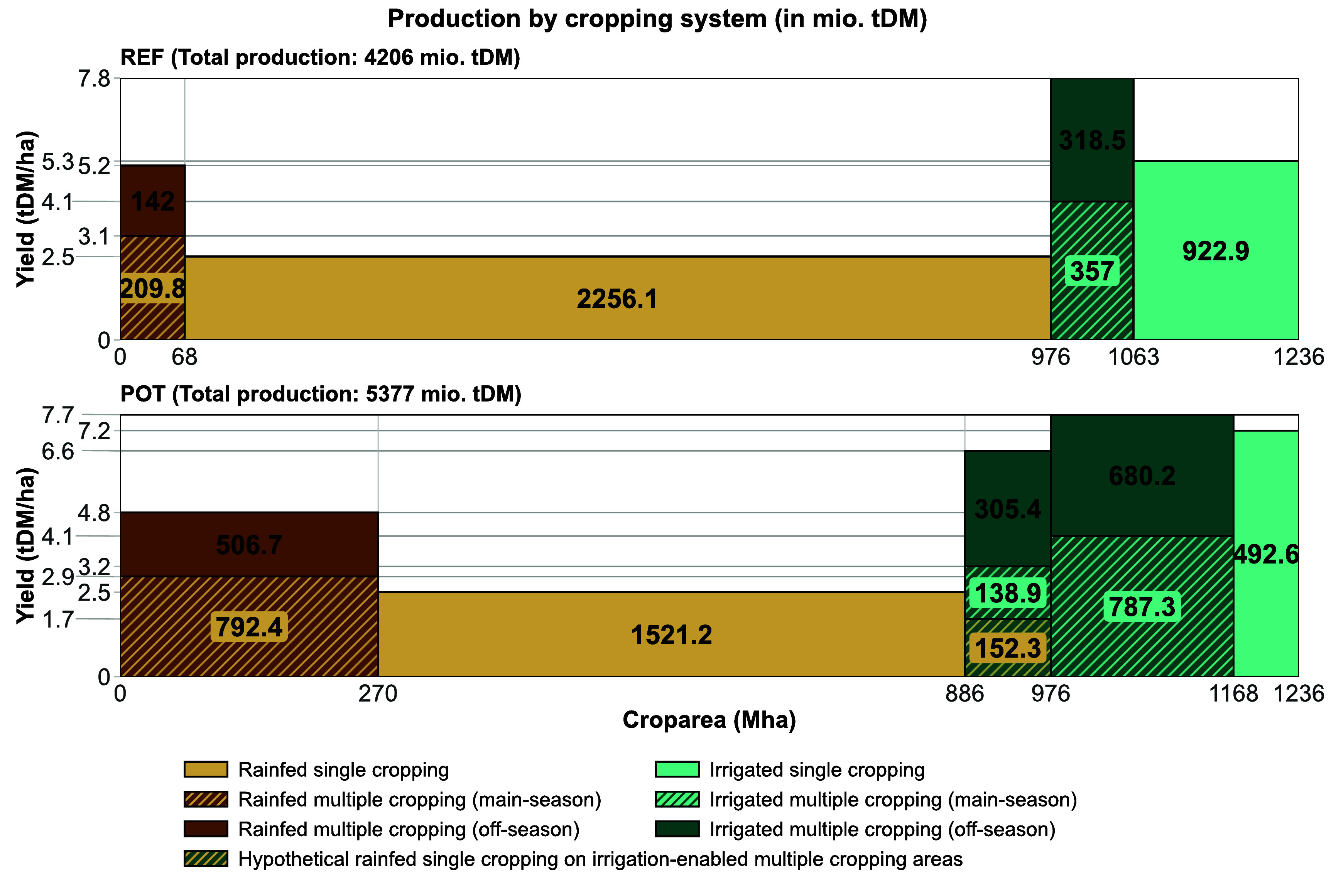
Crop production potential (in million tons dry matter) by area and management system (rainfed multiple cropping, rainfed single cropping, irrigated multiple cropping, irrigated single cropping) and the counterfactuals (main season rainfed and irrigated) for the scenarios REF (distribution of cropping systems on actively cropped physical cropland area as of 2010) and POT (multiple cropping expansion potential considering local water availability constraints).

Figure 3 shows the global production potential on existing cropland by cropping system for the REF and POT scenarios. Total crop production could increase by almost 1 200 mio. t DM (from 4 200 mio. t DM in REF to 5 400 mio. t DM in POT) by expanding multiple cropping on existing cropland.

In REF, we find total crop production of roughly 4 200 mio. t DM. While only roughly 20% of cropland (260 Mha) are under irrigated management, they make up for 38% of global production (almost 1 600 mio. t DM ha^−1^). This shows the important role irrigation plays in facilitating higher yields compared to rainfed crop production—both for single cropping (with irrigated yields of 5.3 t DM ha^−1^ and rainfed yields of 2.5 t DM ha^−1^) and multiple cropping systems (with irrigated yields of 7.8 t DM ha^−1^ and rainfed yields of 5.2 t DM ha^−1^).

An expansion of multiple cropping areas to their biophysical potential (POT) would facilitate total production of almost 5 400 mio. t DM. This is an increase of almost 1 200 mio. t DM (28%). Rainfed production could increase from 2 610 mio. t DM to 2 970 mio. t DM through the expansion of rainfed multiple cropping by 200 Mha, despite slightly lower average yields (dropping from 5.2 to 4.8 t DM ha^−1^) as expansion incorporates less productive areas while the most productive areas are already multiple cropped. On irrigated areas, a multiple cropping expansion would lead to additional production of 360 mio. t DM. Additionally, an expansion of irrigation into areas where irrigated multiple cropping is possible, but under rainfed conditions only one single season is feasible (i.e. areas where irrigation facilitates at least one additional growing season), would add 440 mio. t DM crop production potential. On these areas, an average multiple cropping yield of 6.6 t DM ha^−1^ can be achieved while irrigation of the main growing season alone would only raise the yield from 1.7 t DM (rainfed single cropping yield) to 3.2 t DM ha^−1^ (yield under irrigated single cropping conditions).

The irrigation-enabled multiple cropping expansion potential has not been previously assessed. Mauser *et al* (2015) estimated the potential biomass production increase (PBPI) associated with multiple cropping expansion on current cropland under current irrigation patterns, providing a suitable benchmark for comparison. Figure 11 in the SI shows the global PBPI using our methodology compared to estimates by Mauser *et al* (2015), FAO-GAEZ (Fischer *et al* 2010, IIASA/FA 2012), and Mueller *et al* (2012). The values reported by Mauser *et al* (2015) correspond to our scenario without consideration of local irrigation water availability constraints (NWL) and our estimates for the comparable scenarios fall within previously-estimated ranges. Accounting for local water availability constraints—which previous estimates did not consider—is crucial to avoid overestimating the multiple cropping expansion potential, particularly for irrigation-enabled expansion. Further details can be found in section 4 of the SI.

## 4. Discussion

Our global assessment reveals substantial biophysical potential for multiple-cropping expansion on existing cropland: global production could rise by nearly 30% – without requiring cropland expansion. In our data representing the state of multiple cropping in 2010, large, untapped multiple cropping expansion potentials exist in North- and Central America, Brazil, Sub-Saharan Africa, India and South-East Asia, suggesting that other constraints exist that explain why multiple cropping is not practiced everywhere where it is theoretically possible.

Beyond biophysical growing conditions, other factors such as labor availability, capital costs, market access, socio-cultural practices and additional risks play a role in the farmers’ cropping decisions (Bennett *et al* 2012, VanWey *et al* 2013, Kaini *et al* 2020). For example, the multiple cropping expansion in China slowed down due to the declining agricultural workforce (Wu *et al* 2018, Yin *et al* 2024). In the United States, higher input costs and insurance premiums discourage adoption (Borchers *et al* 2014, Kasu *et al* 2019), while adoption among small-scale farmers in Costa Rica is hindered by the potential risk of crop failure due to off-season weather variability when shifting their growing season outside the main growing window (Radulovic 2000). Across Sub-Saharan Africa and Asia, market access to seeds and other inputs and selling prices for second season crops also influence farmers’ cropping choice (Ekepu and Tirivanh 2016, Yap *et al* 2017, Kaini *et al* 2020). Especially in Africa, parts of Brazil and Southeast Asia, irrigation expansion could facilitate another growing season. This would require both public investments to build canals and dams as well as investments by farmers for on-field equipment or additional labor (Inocencio *et al* 2007), but also facilitate higher returns to these investments.

From a land-sparing perspective, increasing the number of harvests per year on existing cropland and therefore avoiding further cropland expansion can mitigate deforestation and reduce pressure on natural ecosystems and biodiversity therein (Wu *et al* 2018, Folberth *et al* 2020). However, intensification can cause environmental degradation, particularly when multiple cropping involves simplified crop rotations with high input demands in terms of fertilizer, herbicides and pesticides (Matson *et al* 1997, Bennett *et al* 2012, Kremen and Mile 2012, Barbieri *et al* 2017) or when irrigation expansion leads to unsustainable freshwater withdrawals (Matson *et al* 1997, Wada and Bierken 2014, Bhatt *et al* 2021, Jain *et al* 2021). When implemented in diversified systems with crop rotations including legumes or conservation practices, it can improve resource use efficiency per unit of land (Xu *et al* 2020, Waha *et al* 2025), enhance soil health and maintain biodiversity (Ladha *et al* 1996, Bado *et al* 2006, Gaba *et al* 2015, Tang *et al* 2024).

Several assumptions, methodological constraints and data limitations should be considered when interpreting our findings (detailed in SI section 2). First, abstracting from intra-annual variability, our method relies on annual water budgets and assumes that irrigation infrastructure (e.g. dams and canals) can supply water when needed during the growing season (Beier *et al* 2023); this may overstate water availability where seasonal storage or conveyance constraints limit timely delivery. Second, while multiple cropping systems often involve different crops in subsequent cycles (Waha *et al* 2020), we only consider additional cycles of the same crop; rotations are not explicitly represented and are only implicitly captured by crop coexistence within the relatively coarse 0.5 ^∘^ grid cells. Third, while we implicitly assume similar productivity constraints across seasons (i.e. the same yield penalty from management constraints for the second season as for the main season) by using the off-to-main season ratio of grass GPP to derive off-season crop yields, our yield gains are likely lower-bound estimates, given the omission of potential benefits from diversified rotations (Yang *et al* 2024, Waha *et al* 2025) and the off-season yield dampening factor of 25%. Fourth, recognizing that temporary fallow land or cover cropping can be essential for soil health and pest control (Bruun *et al* 2006, Styger *et al* 2007, Hiernaux *et al* 2009, Siebert *et al* 2010), we assess multiple cropping only on actively cropped areas (1 235 Mha), excluding 295 Mha of fallow land that could offer additional intensification potential, which again tends to understate multiple cropping production gains. Moreover, the REF scenario likely underestimates current multiple cropping because of spatial averaging where multiple cropping coexists with fallow land within the same 0.5^∘^ grid cells. Despite these simplifications, the multiple cropping areas in the REF scenario are comparable to other global estimates (Waha *et al* 2020), our crop-specific suitability aligns with multiple cropping zones in GAEZ v.4 (FAO 2022), and our PBPI matches estimates reported in Mauser *et al* (2015).

Our analysis provides a global assessment of the biophysical potential for expanding multiple cropping on existing cropland, but does not account for socio-economic or sustainability constraints, nor future global change. For instance, while we find irrigation-enabled multiple cropping in parts of Brazil, these same regions have already experienced severe droughts in the recent past (Cunha *et al* 2019, Stríkis *et al* 2024). Future research should incorporate sustainability aspects by considering environmental flow requirements to avoid exacerbating water stress (Pastor *et al* 2014, Rosa *et al* 2018, Mehta *et al* 2024), account for climate change impacts such as shifting rainfall patterns and drought risk (Rosa and Sangiorgi 2025), and dive deeper into the individual crop level, for example also including crop rotations or diversified multiple cropping systems (Waha *et al* 2020). Accounting for multiple cropping is crucial for estimating the spatial distribution of crop production and irrigation water use, forming the basis for a wide range of global assessments from climate impact and hydrological studies to those evaluating intensification and land-use change (Waha *et al* 2025). It is particularly relevant for subtropical and tropical regions where multiple cropping and irrigation are widespread (Biemans *et al* 2016, Waha *et al* 2020), and is also relevant for global-scale applications, for which the generic approach presented here can be applied.

## 5. Conclusion

This study presents the first global assessment of potential crop production gains from intensifying existing cropland through irrigation-enabled multiple cropping while accounting for local water availability constraints. Annual production could rise by roughly 28% without cropland expansion, with the largest opportunities in Brazil, Sub-Saharan Africa, India and South-East Asia. Local freshwater availability constrains this potential, underscoring the importance of jointly considering multiple cropping and irrigation.

## Data Availability

The data that support the findings of this study are openly available at the following URL/DOI: The open-source code of mrwater v.1.13.7.9012 used for this publication is available on GitHub (https://github.com/FelicitasBeier/mrwater/releases/tag/v1.13.7.9012) (Beier *et al* 2025). The source code is also stored on Zenodo (https://doi.org/10.5281/zenodo.17292996) together with the input data to the algorithm including LPJmL data, LandInG data and mappings (see version v2 under https://doi.org/10.5281/zenodo.18430142) as well as the output data and the scripts used to create tables and figures for this publication (see version v.1.0 under https://doi.org/10.5281/zenodo.17292997) (Beier *et al* 2026). Supplementary Material available at https://doi.org/10.1088/1748-9326/ae44ae/data1.
